# Dietary intake, anthropometric measurements, biochemistry profile and their associations with chronic kidney disease and diabetes mellitus

**DOI:** 10.1017/jns.2020.38

**Published:** 2020-09-30

**Authors:** Emily de S. Ferreira, Luciana S. da Silva, Glauce D. da Costa, Tiago R. Moreira, Luíza D. Borges, Rosângela M. M. Cotta

**Affiliations:** 1 Department of Nutrition and Health (DNS), Federal University of Viçosa (UFV), Viçosa, MG, Brazil; 2 Department of Nutrition, Federal University of Uberlândia (UFU), Uberlândia, MG, Brazil; 3 Department of Medicine and Nurse, Federal University of Viçosa (UFV), Viçosa, MG, Brazil

**Keywords:** Renal insufficiency chronic, Primary health care, Dietary intake, Anthropometry, Biomarkers

## Abstract

The chronic kidney disease (CKD) and diabetes mellitus (DM) are considered a serious public health problem. The objective was investigating the association of DM with the anthropometric measures, biochemical profile and dietary intake in patients with CKD. Is a cross-sectional study done in 2017, with 51 patients previously diagnosed with CKD. We collect socio-demographic, lifestyle variables, anthropometric measurements, biochemical profile and dietary intake. We using the Kolmogorov–Smirnov test, followed by Pearson's *χ*^2^ test and Student's *t* test. Data were analysed using several multivariable logistic regression models, including the socio-demographic, anthropometric, dietary intake and biochemical variable. Variables with *P* ≤ 0⋅20 in the univariate analyses were selected and kept in the block in the simple and multiple logistic regression analysis, to determine the differences between the categories and the factors associated with the presence of DM or not, remaining in the model final, only the significant variables (*P* ≤ 0⋅05). Each variable was adjusted for all other variables included in the univariate analysis. The strength of the association was assessed by the odds ratio and 95% confidence intervals (CI). The multivariate logistic regression analysis evidenced that the increase of 1 cm in waist circumference and 1 mg/dl in VLDL-c values increases the chance of DM, respectively, by 8⋅4% (OR 1⋅076; *P* 0⋅05) and 8⋅8% (OR 1⋅102; *P* 0⋅01). In contrast, an increase of 1 mg/dl in total cholesterol decreases the chance of developing DM by 3⋅1% (OR 0⋅965; *P* 0⋅01), that is, it becomes a protective factor. The present study identified the associations between overweight, dietary intake and biochemical tests.

## Introduction

The prevalence of chronic kidney disease (CKD) expands globally, with 8–16 % annual increase, a rate higher than the general population growth^(1)^. CKD is considered a serious public health problem. Currently, 52 million people are at risk of developing CKD because of advanced age, obesity, inadequate eating habits and a sedentary lifestyle, hypertension (AH) or diabetes mellitus (DM). Conditions that make these groups more prone to kidney problems^(1)^.

Diabetes is highly prevalent worldwide. It is frequently associated with AH, population ageing, inadequate eating habits and CKD development^(1,2)^. When these factors are combined and there is prolonged exposure to high glucose blood levels, it can lead to diabetic nephropathy, a disease affecting 30 % of diabetics^(1)^.

CKD is asymptomatic most of the time, with a long and insidious course characterised by the progressive and irreversible loss of the endocrine and glomerular functions, and consequently, renal functions^(3)^. CKD is featured by an individual who has a glomerular filtration rate (GFR) <60 ml/min/1⋅73 m^2^, or >60 ml/min/1⋅73 m^2^ associated with one marker of renal parenchymal damage for three consecutive months, at least.

Inadequate dietary intake, and weight gain, have a strong influence on CKD development^(4,5)^. These modifiable factors are poorly monitored by patients and health professionals in the primary health care system, and yet directly influence other chronic diseases, such as AH.

Primary health care should be focused on strengthening prevention, including nutritional orientation for people with CKD^(6)^. An adequate and individualised diet for each patient, coupled with other nutritional assessment parameters, including biochemical and anthropometric measures (e.g. height, weight, Body Mass Index – BMI, Waist Circumference – WC), can significantly delay the onset of the disease or mitigate the deleterious effects in its course^(2)^.

Thus, the objective of the present study is to investigate the association of DM with anthropometric, biochemical and dietary intake profile in CKD patients.

## Methods

### Participants

113 patients previously diagnosed with CKD, which were enrolled in and monitored by the Primary Health Care unit of the city of Porto Firme, State of Minas Gerais, Brazil, were invited to participate in this cross-sectional study carried out in 2017. Fifty-one patients agreed to participate. CKD was assessed by estimating GFR with the CKD-EPI formula and by 24-h proteinuria and microalbuminuria urine collection.

Diabetic patients were evaluated with several parameters, including (1) fasting glucose and glycated haemoglobin blood levels; (2) anthropometric profile according to weight, BMI and WC; (3) lipid profile biochemical analysis; (4) dietary intake assessment based on patients’ daily diet recall, and also based on a questionnaire of food consumption frequency validated for the adult population and adapted for the present study.

### Data collection

We classified BMI according to the WHO adults’ criteria^(7)^, and Lipschitz's (1994) criteria for the elderly^(8)^, the same cutoff points were used to predict nutritional status. WC was classified according to the cutoff points proposed by the WHO^(7)^, the same cutoff point was used to predict mild, moderate or high risk of metabolic complications. In relation to dietary intake, we analysed macronutrients (carbohydrates, proteins and lipids) and micronutrients (calcium, phosphorus, potassium and sodium). We also collected demographic, lifestyle and health care variables. We chose to use a semi-structured interview script as a tool to collect information on the variables analysed. Daily diet recall was applied by properly trained researchers with the help of a photographic album including the most common dishes for the Brazilian population. *Per capita* consumption of salt, oil and sugar was estimated by the duration of one kilo/litre of these items, and the number of residents in the patients’ house.

### Ethics Committee and formal consent

The present study was conducted by following the guidelines laid down in the Declaration of Helsinki and all procedures involving human subjects/patients were approved by the Human Research Ethics Committee of the University, under protocol number 1.139.717/2015. Written informed consent was obtained from all subjects/patients.

### Statistical analyses

Qualitative variables were presented in tables of absolute and relative frequency, while quantitative variables were described with measures of central tendency (mean), dispersion (maximum value, minimum value, standard deviation – sd and variance) and frequency (absolute and relative).

All continuous variables’ distribution was tested with the Kolmogorov–Smirnov test, followed by Pearson's *χ*^2^ test to compare independent categorical variables, and Student's *t* test to investigate the presence of DM and its relationship with the variables of interest. An initial model of multivariate regression was built then, including socio-demographic, anthropometric, biochemical and dietary intake variables.

In order to determine the differences between the categories and the factors associated with DM presence, our dependent variable, all the variables with *P* < 0⋅20 in the univariate analysis were selected and maintained *en bloc* in the simple and multiple logistic regression analyses. Each variable was adjusted for all other variables included in the univariate analysis (gender, WC, BMI, total cholesterol, oil consumption, glucose, HDL-c, LDL-c, VLDL-c and triglycerides). The multiple logistic regression analysis was progressively refined by the Wald test, only significant variables remained on the final model (*P* ≤ 0⋅05). The strength of the association was assessed by the odds ratio (OR) and 95 % confidence intervals (CIs).

Dietpro software version 5⋅8 was used to analyse dietary intake data. All statistical analyses were conducted on SPSS Statistics version 20.0 (IBM, SPSS Inc, Chicago) for Windows. All statistical tests were two-tailed and *P* values ≤ 0⋅05 were considered statistically significant.

## Results

**Fig. 1.**
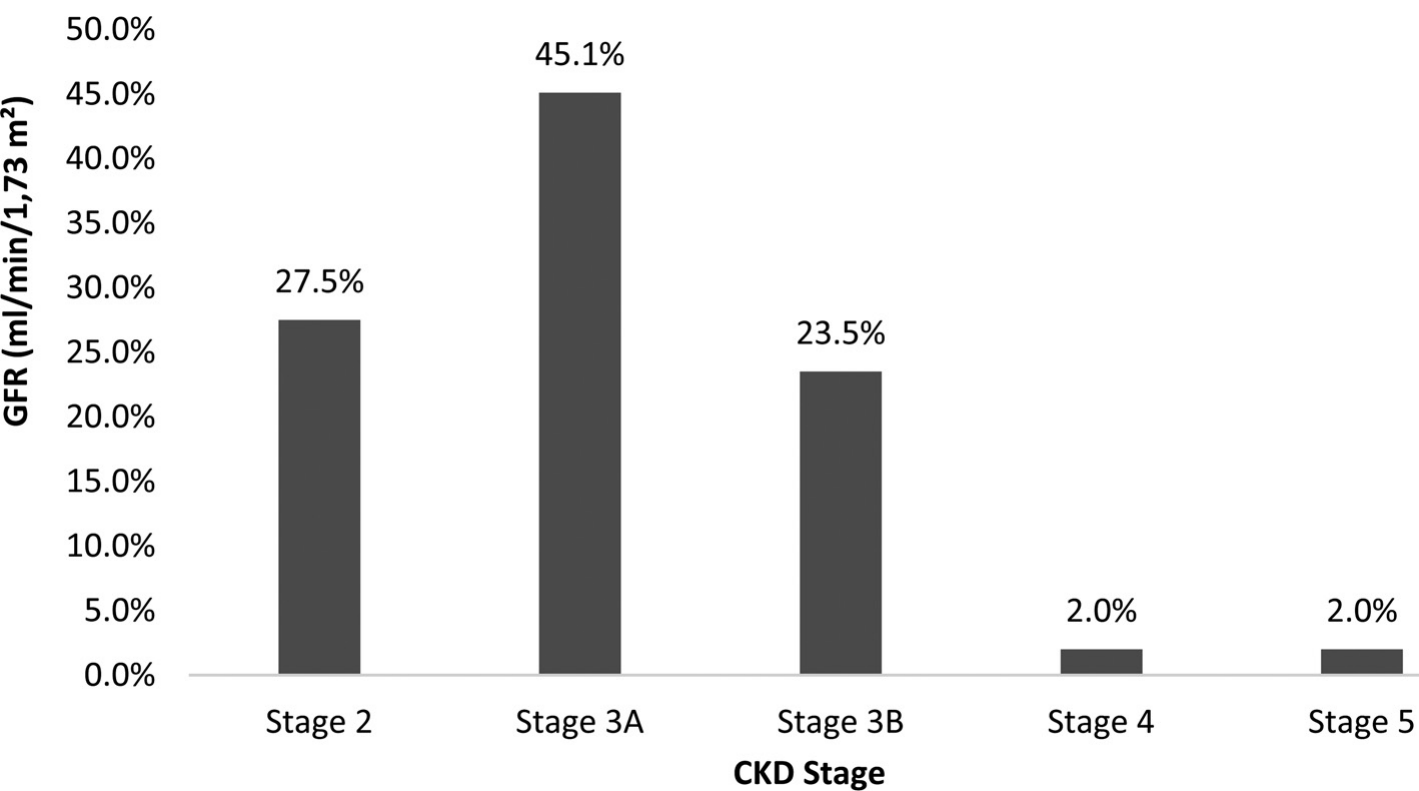
Stages of chronic renal disease according to the glomerular filtration rate.

Regarding GFR, the majority of patients (45⋅1 %) were in the disease stage 3a (GFR of 45 to 59 ml/min/1⋅73 m^2^) (Fig. 1), and mean GFR was 53 ml/min/1⋅73 m^2^.

Table 1 shows the socio-economic characteristics and anthropometric characteristics of the patients studied. Of the fifty-one individuals participating in the study, thirty-seven (72⋅5 %) are female. The majority are in the 76–85 years old age group (45⋅1 %), are retired (86⋅3 %) and with low schooling (43⋅2%).

**Table 1.** Socio-economic and anthropometric characteristics of CKD patients evaluated

Total number of participants = 51	*n* (%)
Sex	
Female	37 (72⋅5)
Male	14 (27⋅5)
Age (Years)	
≤65	7 (11⋅9)
≥66	44 (74⋅6)
Education	
Illiterate	16 (31⋅4)
Read and write	6 (11⋅8)
1 and 4 years	22 (43⋅1)
5 year to higher education	7 (13⋅7)
Occupation	
Informal work	7 (13⋅7)
Retired/pensioner	44 (86⋅3)
Family income	
<1 minimum wage	1 (2⋅0)
1 and 3 minimum salaries	45 (88⋅2)
3 and 5 minimum wages	5 (9⋅8)
Marital status	
Without partner	22 (43⋅2)
With partner	29 (56⋅9)
Nutritional status	
Low weight	8 (15⋅7)
Eutrophic	16 (31⋅4)
Overweight	27 (52⋅9)
Risk of metabolic complications	
Without risk	12 (23⋅5)
Increased risk	8 (15⋅7)
Substantially increased risk	31 (60⋅8)

Table 1 presents the patients’ socio-economic and anthropometric characteristics. Thirty-seven individuals participating in the study were females (72⋅5 %). The majority were in the 76–85 years old age group (45⋅1 %), were retired (86⋅3 %) and with low scholarity (43⋅2 %). Most patients never smoked (64⋅7 %), fourteen were ex-smokers (27⋅5 %) and most of them do not consume alcohol (86⋅3 %). 35⋅3 % of the patients were diabetic. Overweight prevalence was 52⋅9 % and risk of metabolic complications increased substantially (60⋅8 %) with greater WC in both genders.

Table 2 presents CKD patients’ socio-economic, anthropometric, dietary intake and biochemical variables according to DM prevalence. Amongst diabetic patients, the average age was 73⋅6 years ± 9⋅0 (mean ± sd) (*P* 0⋅173). Regarding the anthropometric measurements, the highest WC (101⋅7 cm ± 8⋅9, *P* 0⋅002) and BMI (30⋅1 kg m² ± 4⋅9, *P* 0⋅022) averages were observed in the group with DM, whereas the average WC was 90⋅9 cm ± 12⋅3 and BMI was 26⋅4 kg m² ± 5⋅6 in the group without DM.

**Table 2.** Socio-economic, anthropometric, dietary intake and biochemical variables of CKD patients according to the presence of diabetes mellitus

Variables	Diabetes		*P* value*
	Yes (Mean/sd)	No (Mean/sd)	
Socio-demographic			
Age (years)			
≤65	3 (5⋅9)	4 (7⋅8)	0⋅652
≥66	15 (29⋅4)	29 (56⋅9)	
With partner	10 (55⋅6)	19 (57⋅6)	0⋅889
Without partner	8 (44⋅4)	14 (42⋅4)	
Sex			
Female	15 (83⋅3)	22 (66⋅7)	0⋅202*
Male	3 (16⋅7)	11 (33⋅3)	
Anthropometric			
WC (cm)	101⋅68 (8⋅9)	90⋅89 (12⋅3)	0⋅002*
BMI (kg/m^2^)	30⋅10 (4⋅9)	26⋅39 (5⋅6)	0⋅022*
Dietary intake			
CHO (% consumption)	62⋅84 (10⋅0)	60⋅3 (14⋅0)	0⋅502
PNT (g consumption)	34⋅6 (28⋅2)	33⋅91 (19⋅6)	0⋅919
LIP (% of consumption)	20⋅40 (6⋅1)	20⋅21 (9⋅6)	0⋅939
Calcium (mg)	317⋅4 (240⋅5)	301⋅06 (190⋅6)	0⋅791
Sodium (mg)	696⋅04 (542⋅3)	551⋅65 (352⋅6)	0⋅255
Potassium (mg)	1395⋅95 (747⋅2)	1206⋅63 (444⋅8)	0⋅261
Phosphorus (mg)	519⋅3 (362⋅2)	504⋅9 (255⋅3)	0⋅869
Water (ml/d)	67⋅17 (38⋅0)	83⋅4 (65⋅2)	0⋅448
Oil (ml/d)	94⋅8 (25⋅8)	118⋅3 (34⋅3)	0⋅017*
Salt (g/d)	9⋅71 (4⋅5)	10⋅3 (7⋅7)	0⋅790
Sugar (g/d)	67⋅17 (38⋅0)	83⋅4 (65⋅2)	0⋅448
Biochemical exams			
Glucose (mg/dl)	119⋅72 (42⋅0)	90⋅82 (28⋅4)	0⋅005*
Total cholesterol (mg/dl)	173⋅06 (25⋅5)	190⋅30 (43⋅4)	0⋅129*
HDL-c (mg/dl)	42⋅83 (6⋅3)	48⋅03 (8⋅7)	0⋅031*
LDL-c (mg/dl)	95 (26⋅0)	118 (34⋅0)	0⋅017*
Triglycerides (mg/dl)	192⋅9 (106⋅0)	119⋅42 (65⋅5)	0⋅004*
VLDL-c (mg/dl)	35⋅12 (15⋅5)	24 (13⋅2)	0⋅010*

WC, waist circumference; BMI, body mass index; CHO, carbohydrate; PTN, protein; LIP, lipid; sd, standard deviation; HDL, high-density lipoprotein; LDL, low-density lipoprotein; VLDL, very-low-density lipoproteins.

* *P* < 0⋅20 by *χ*^2^ test or Student's *t* test.

Furthermore, macronutrients intake was within the recommended values for this population, in both groups. Micronutrients (calcium, sodium, potassium and phosphorus) consumption, however, was higher in patients with DM than patients without DM. Sodium intake was within the recommended level (696⋅04 mg ± 542⋅3, *P* 0⋅255). Other micronutrients were below recommended levels in both groups.

*Per capita* consumption of oil, sugar and salt was higher in the group without DM. The average salt intake was 9⋅71 g/d ± 4⋅5 for patients with DM and 10⋅3 g/d ± 7⋅7 for patients without DM (*P* 0⋅790).

Biochemical analyses, as expected, showed high fasting glucose levels in the DM population (119⋅72 mg/dl ± 42⋅0, *P* 0⋅005). Triglycerides and VLDL-c values were also significantly elevated in patients with DM (192⋅9 mg/dl ± 106⋅0 and 35⋅12 mg/l ± 15⋅5, respectively; *P* 0⋅004).

Multiple logistic regression analysis was conducted to explore the factors related to DM presence (Table 3). Based on the univariate analyses results, the following variables were included in the multiple logistic regression model: WC, BMI, *per capita* oil consumption, total cholesterol, HDL-c, LDL-c, triglycerides and VLDL-c. According to the gross and adjusted analyses, the parameters WC, total cholesterol and VLDL-c were independently associated with DM.

**Table 3.** Logistic regression analysis on associations between socio-economic, anthropometric, dietary intake and biochemical variables of patients with CKD according to diabetes mellitus^a^

Variables	Crude analysis			Adjusted analysis		
	OR	95 % CI	*P*	OR	95 % CI	*P*
Óil (ml)	0⋅982	0⋅95–1⋅01	0⋅24	0⋅99	0⋅95–1⋅03	0⋅75
WC (cm)	1⋅094	1⋅03–1⋅17	0⋅05	1⋅076	1⋅00–1⋅16	0⋅05
Total cholesterol (mg/dl)	0⋅986	0⋅97–1⋅00	0⋅13	0⋅965	0⋅94–0⋅99	0⋅01
Glucose (mg/dl)	1⋅025	1⋅00–1⋅05	0⋅02	1⋅007	0⋅98–1⋅03	0⋅53
BMI (kg/m^2^)	1⋅136	1⋅01–1⋅27	0⋅03	1⋅124	0⋅83–1⋅52	0⋅45
HDL-c (mg/dl)	0⋅909	0⋅83–0⋅99	0⋅04	1⋅043	0⋅92–1⋅12	0⋅52
LDL-c (mg/dl)	0⋅972	0⋅95–0⋅99	0⋅02	1⋅124	0⋅81–15⋅64	0⋅93
Triglycerides (mg/dl)	1⋅011	1⋅00–1⋅02	0⋅01	1⋅042	0⋅62–1⋅74	0⋅87
VLDL-c (mg/dl)	1⋅055	1⋅00–1⋅10	0⋅02	1⋅102	1⋅02–1⋅18	0⋅01
Sex						
Female	1	–	0⋅68	1		–
Male	0⋅400	0⋅09–1⋅68	0⋅21	0⋅460	0⋅46–4⋅59	0⋅50

OR, odds ratio; CI, confidence intervals; WC, waist circumference; BMI, body mass index; HDL, high-density lipoprotein; LDL, low-density lipoprotein; VLDL, very-low-density lipoproteins.

a Multiple logistic regression analysis. Each variable was adjusted for all other variables in the table. The dependent variable is the presence of the DM.

Both WC and VLDL-c levels were positively associated with DM development, 1 cm increases in WC measure made patients 8⋅4 % more likely to develop DM (OR 1⋅076; *P* 0⋅05), and 1 mg/dl increase in VLDL-c values raises the chance of DM development in 8⋅8 % (OR 1⋅102; *P* 0⋅01). High levels of total cholesterol, on the other hand, were negatively associated with DM development, 1 mg/dl increase in blood level decreases the chance of developing DM by 3⋅1 % (OR 0⋅965; *P* 0⋅01), that is, it becomes a protective factor.

## Discussion

The present study aspired to investigate the association between DM and anthropometric, biochemical and dietary intake profiles in patients diagnosed with CKD living in Porto Firme region, Brazil. Our main finding was that WC (101⋅68 cm mean), total cholesterol (173⋅06 mg/dl mean) and VLDL-c (35⋅12 mg/dl mean) were correlated with DM development during CKD. The present study demonstrated that DM was prevalent among 35⋅3 % of the patients with CKD. Although DM is a known risk factor for CKD, some of the patients diagnosed with CKD were unaware of the presence of DM or did not treat the disease.

All diabetic patients in our study had CKD, similarly, in a study by Francisco *et al.*^(9)^ CKD prevalence was about three times higher amongst diabetic elderly than non-diabetic patients. CKD and DM association can be explained by the greater predisposition of diabetics to develop some kind of nephropathy, which can lead to chronic kidney failure, and further, haemodialysis or kidney transplantation, given that CKD is resistant to anabolic hormones, such as insulin and growth hormones.

Regarding the anthropometric data, Chi-yuan *et al*.^(10)^ observed that high BMI and WC are major risk factors for CKD and DM since overweight and obesity are known to be predisposing to these diseases. Their progression has increased the rate of kidney function loss. Amador *et al*.^(11)^ observed that abdominal obesity, based on WC measures, was positively associated with GFR. There are several possible explanations for the influence of the main disease risk factors in CKD, including DM and AH, being that the increase in BMI, and especially WC, are proportional to the increase in CKD^(12)^. Secondly, overweight individuals have a more developed adipose tissue in their renal capsule, which compresses glomerular systems and tubular filtration, increasing the GFR and, consequently, increasing tubular sodium reabsorption^(4,13)^.

There is a difference in body composition in men and women, these differences being observed in WC and BMI at all ages and levels of fat, which is the reason the cutoff points are different for analysing WC between men and women^(14)^. Among women, parity and menopause have hormonal changes, which have a strong association with changes in body composition^(14)^. Men, in turn, have greater muscle mass, bigger and stronger bones and reduced fat in the limbs. Coupled with all this, we have the ageing factor, which is associated with the substantial redistribution of adipose tissue between deposits and has a direct implication on the body composition of both, men and women^(15)^.

VLDL-c (mg/dl) and total cholesterol significant correlation with DM development in this study may be related to the excessive consumption of high-fat foods. The lipotoxicity of free fatty acids in the body leads to kidney lesions, through the deregulated accumulation of triglycerides transported by VLDL-c in non-adipose tissues, such as the kidney tissue, blocking cell signalling pathways and inducing apoptosis^(16)^.

In contrast with the previous literature, total cholesterol (mg/dl) was negatively correlated with DM development^(17–19)^. Our finding lines up with the study by Kovesdy *et al.*^(20)^, who observed that in patients receiving conservative treatment, serum cholesterol in the low normal range levels (<150–180 mg/dl) were related to higher mortality than high cholesterol levels. We can only speculate the reason for this discrepancy concerning the role of cholesterol in DM development in patients with CKD, across different studies. Decreased lipoprotein lipase activity caused by insulin resistance increases triglycerides levels in the blood^(21)^, as well as the average triglycerides levels in diabetic patients. High triglycerides levels, rather than cholesterol, may contribute more severely to cardiovascular diseases, which are the major causes of mortality among patients with CKD according to Takenaka *et al.*^(22)^ and Scarpione *et al.*^(21)^. Thus, to clarify why total cholesterol has protective effects on DM development in some populations, but not in others, more detailed studies will be required, with wider dietary assessment, or studies with biomarkers.

In addition, was an interaction described by Iseki *et al*.^(23)^ in which higher total cholesterol leads to complications only in patients with high serum albumin levels and in high stages of CKD. As a result, these results suggested that lower total cholesterol is a substitute marker for inflammation and/or malnutrition, which are also complicating CKD. Despite this, therapy aimed at reducing cholesterol should not be withheld, especially considering the additional effects, potentially unrelated to the use of statins (the most widely used cholesterol-lowering drugs)^(24,25)^.

The protective effect of total cholesterol on DM could be explained by the use of statins by patients; however, we cannot confirm this point of view.

Our results indicate that the average phosphorus intake based on the daily dietary intake recall was 1395⋅95 mg ± 747⋅2 in the group with diabetes and 1206⋅63 mg ± 444⋅8 in the group without diabetes, both within the recommended level for this population, similar to the findings of Cabral *et al*.^(26)^.

The average calcium intake of 317⋅4 mg ± 240⋅5 was similar to the results of Cabral *et al.*^(26)^ and Valenzuela *et al*.^(27)^, in which both groups did not reach the recommended intake level. Foods richer in phosphorus are rich in calcium as well. Although changes in phosphorus and calcium metabolism are not frequent in the early stages of CKD, which is the reality of the population studied, their consumption should be maintained in the normal range to prevent other complications, such as hypercalcemia and hyperphosphatemia, which may lead to calcium deposition in soft tissues and CKD worsening.

All the individuals analysed based on the usual dietary intake recall consume sodium within the recommended level. In the diabetic group, the average intake was 696⋅04 mg ± 542⋅3 and in the non-diabetic group, it was 551⋅65 mg ± 352⋅6. However, when analysing salt intake and its monthly consumption, the amount of 9⋅71 g/d ± 4⋅5 in the diabetic group exceeded the recommended value of 5 g/d proposed by the WHO^(7)^ for the general population. In the present study, all meal analysis was performed in the home environment.

Sodium daily consumption is above 2⋅3 g in several countries, WHO recommends between 2 and 5 g/d.

## Strengths and limitations

The strengths of our study included results would be useful for health professionals to identify the high-risk individuals, for efficient screening of DM at the primary health care level. This information would also assist the health authorities and policymakers to develop specific screening guidelines based on obesity indicators that would best identify high-risk individuals in the community for appropriate intervention. Our study also showed that not all diabetics, as well as kidney patients, knew about the presence of diseases. We have to consider that not all patients had dietary changes before the start of the study. In addition, some patients reported that they knew they were diabetics but did not follow all the guidelines given.

However, our study had limitations. First is a cross-sectional study, and we cannot draw conclusions about cause-and-effect between dietary patterns and various health parameters. Second, our participants also came from that of a specific region of Brazil and, therefore, may not be representative of the Brazilian population; therefore, the results may not be generalised to the general population. Third, self-report of dietary intake by questionnaire is a crude method and constitutes a well-known weakness due to recall bias and social desirability bias, which means that there is an obvious risk of misclassification of the number of portions consumed. It is also important to mention or place limitations for not having assessed body composition by bioimpedance or dual-energy X-ray absorptiometry (DXA), in order to measure the agreement between them. In addition to the fact that we did not have data on the use of medications, especially the statin and the level of physical activity of the population studied since a specific questionnaire for this type of analysis would be necessary.

## Future research

While the protective role of healthy eating against DM was confirmed by our study, the role of the relationship between food consumption and biochemical parameters in the impact of these chronic disease's development is still in question and needs more research. Furthermore, it seems important to investigate if specific types of food are more protective than others by investigating mechanisms of action. To do that a food diary and ideally blood samples would be required in order to analyses nutritional biomarkers of intake^(28)^.

Future research should also aim at developing and evaluating interventions which will increase the intake of foods that improve the significant markers for DM and CKD in the population, and especially among the elderly.

## Conclusion

In conclusion, the results of our study suggest that WC and lipid profile were associates with DM and CKD, being that the increase in VLDL-c increases the chances of the occurrence of DM and the increase in total cholesterol implies in a decrease in the chance of occurrence of the disease. In order to clarify the mechanisms behind these differential effects of total cholesterol, more research is needed with a more detailed assessment of the impacts of the lipid profile for this population. In addition, among diabetics, the WC and BMI index were higher when compared to individuals without DM. The utility of the WC in clinical practice may better stratify at-risk patients in this population than BMI, which is widely used at present.

We also emphasise that the majority of our sample consists of elderly individuals, overweight and in stage 3A of CKD, which requires greater attention and monitoring when eating food. In addition, as noted, BMI and WC are highly correlated, and gender and age associations with BMI, body composition and fat deposit distribution are relevant to WC.

Individuals with a less healthy lifestyle were more likely to have less favourable dietary patterns, and this may possibly lead to a higher risk of obesity, DM and CKD. The influence of demographic, anthropometric, lifestyle and biochemicals characteristics on dietary patterns in the present study provides insights for the tangible dietary advice and primary health care with guidelines to the routine of people with these risk factors or illnesses.

This demonstrates the role of primary health care in the prevention of chronic diseases and their complications through frequent monitoring of the at-risk population since prevention is the best way to control modifiable risk factors.
